# A Threshold of Objectively-Assessed Daily Sedentary Time for All-Cause Mortality in Older Adults: A Meta-Regression of Prospective Cohort Studies

**DOI:** 10.3390/jcm8040564

**Published:** 2019-04-25

**Authors:** Po-Wen Ku, Andrew Steptoe, Yung Liao, Ming-Chun Hsueh, Li-Jung Chen

**Affiliations:** 1 Graduate Institute of Sports and Health, National Changhua University of Education, Changhua City 500, Taiwan; powen@cc.ncue.edu.tw; 2 Department of Sports Science, National Tsing Hua University, Hsinchu City 300, Taiwan; 3 Department of Behavioural Science and Health, University College London, London WC1E 6BT, UK; a.steptoe@ucl.ac.uk; 4 Department of Health Promotion and Health Education, National Taiwan Normal University, Taipei 106, Taiwan; liaoyung@ntnu.edu.tw; 5 Department of Physical Education, National Taiwan Normal University, Taiwan. No.162, He-ping East Road, Section 1, Taipei 106, Taiwan; 6 Department of Exercise Health Science and Graduate Institute of Recreational Sport Management, National Taiwan University of Sport, Taiwan. No. 16, Section 1, Shuang-Shih Rd., Taichung 404, Taiwan

**Keywords:** sedentary behavior, sitting, inactivity, review, cut-point, recommendation, meta-analysis

## Abstract

*Background:* This meta-analysis aimed to estimate the shape of the dose-response association between objectively-assessed daily sedentary time (ST) and all-cause mortality, and to explore whether there is a threshold of ST above which there is an increase in mortality risk in older adults. *Methods:* Searches for prospective cohort studies providing effect estimates of daily ST (exposure) on all-cause mortality (outcome) were undertaken in five databases up to 31 March 2019. A random-effects meta-regression model was conducted to quantify the dose-response relationship between daily ST and all-cause mortality. Sensitivity analyses were also performed to test the stability of the results. *Results:* Our analysis of pooled data from 11 eligible studies did not reveal a consistent shape of association between ST and mortality. After excluding three studies with potential confounding bias, there was a log-linear dose-response relationship between daily ST and all-cause mortality. Overall, higher amounts of time spent in sedentary behaviors were associated with elevated mortality risks in older adults. Visual assessments of dose-response relationships based on meta-regression analyses indicated that increased mortality risks became significant when total ST exceeded approximately 9 h/day. *Conclusions:* Based on a limited number of studies, this meta-analysis provides a starting point for considering a cut-off of daily sedentary time, suggesting older adults spend less time in daily sitting.

## 1. Introduction

Sedentary behaviors such as television viewing, reading, computer/tablet use, passive transport, and sitting and lying down are common among older adults. A systematic review based on 11 large-scale population studies/surveys using objective accelerometry measures reported that older adults spend approximately 10 h a day in sedentary behaviors, equating to 65–80% of their waking day [1]. Unfortunately, sedentary time (ST) has been increasingly recognized as an independent risk factor for different health outcomes in later life [2], and is estimated to be responsible for 3.8% of all-cause mortality in middle-aged and older adults (aged 40–79) [3]. Prolonged sitting has been acknowledged as a serious issue in public health recommendations [4,5,6], which suggests all adults (including older adults) should reduce the amount of daily ST.

Previous reviews have argued that higher amounts of ST are associated with increased risks of all-cause mortality in older adults [7], but a recent systematic qualitative review of prospective studies with objectively-assessed measures reported inconsistent results [2]. Several cohort studies in this later review did not show a significant association between objectively-measured ST and all-cause mortality in older adults [8,9]. The inconsistent findings may be due to heterogeneity across studies. The studies included in the two earlier reviews were primarily based on self-report ST instead of device-based measures. Self-reported measures such as questionnaires are vulnerable to recall bias, leading to less accurate estimates [10]. Additionally, accelerometer wear time (or standardizing wear time for each participant) [11] and moderate-to-vigorous physical activity (MVPA) can confound analyses of ST and mortality [12,13]. However, four cohort studies examining associations between objectively-assessed ST and mortality did not include these factors [8,9,14,15] and three did not observe significant relationships [8,9,14]. Therefore, the inconsistent findings among studies with device-based ST assessments in older adults may be due to confounding effects that deserve further investigation.

A recent narrative review suggested that the dose-response association of daily ST and most long-term outcomes is not linear [6]. The cut-off of daily ST for elevating death hazards in studies with device-based ST may be in the region of 8–9.5 h a day. In contrast, studies with self-reported ST may underestimate sedentary time (40–60%) by a relatively large amount of measurement errors [6]. However, these findings were derived from adult general populations rather than older populations. It is possible that the prevalence, patterns and contexts of sedentary behaviors in adults of working age are distinct from those in later life. To date, there is no evidence from meta-analysis evaluating the relationships of ST and all-cause mortality in older adults based on studies using device-based measures of sedentary behavior.

To fill this gap in the literature, our review adopted meta-regression analyses to explore the dose-response relationship between daily ST and all-cause mortality in older adults aged 65 or older based on well-designed prospective cohort studies using objective device-based assessment, and to examine whether there is a threshold of ST above which health is impaired in older adults. We also conducted sensitivity analyses (e.g., excluding studies with potential confounding bias and investigating underlying moderators of observed associations) to test the robustness of the findings.

## 2. Experimental Section

### 2.1. Study Selection

**Figure 1:**
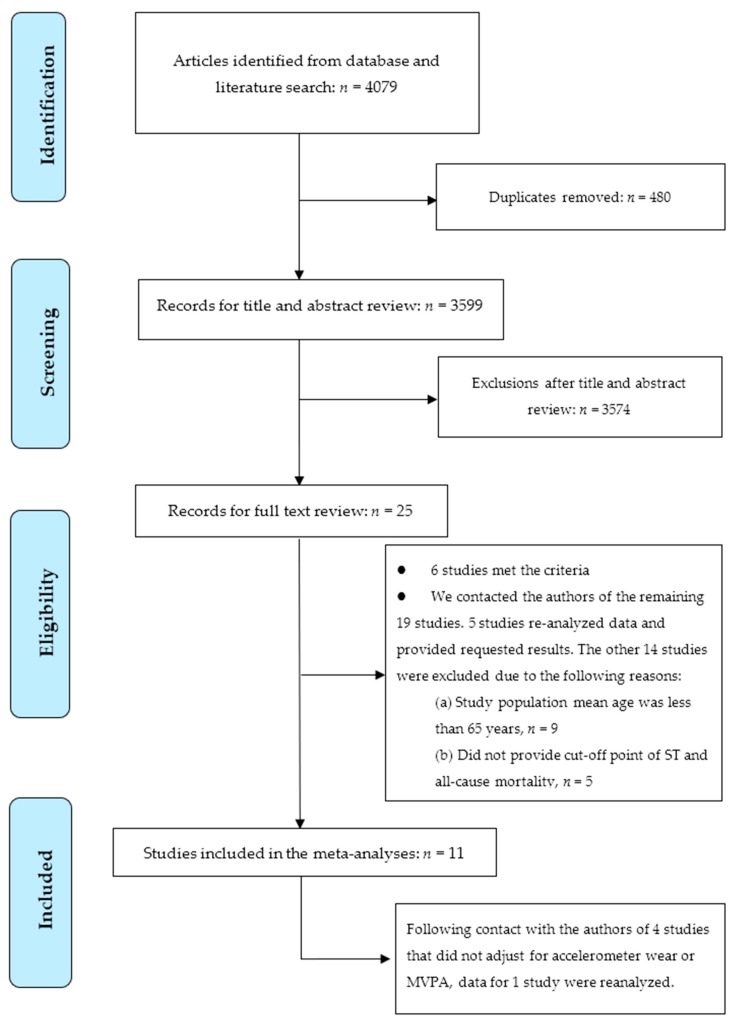
PRISMA diagram showing study selection.

Following the guidelines of Preferred Reporting Items for Systematic Reviews and Meta-Analyses [16], searches for potential articles were undertaken in five electronic bibliographic databases (PubMed, Medline, Scopus, EMBASE, and Web of Science) up to 31 March 2019. The following search terms [2]: (elderly OR “older adults” OR “old people” OR “elders” OR “aged” OR “senior citizens” OR “retired” OR “retirees”) AND (sitting OR sedentary OR sedentariness OR sedentarism OR “sedentary behaviour” OR “sedentary behavior”) AND (mortality OR mortalities OR death OR fatal) AND (risk OR Cox OR hazard OR odds) AND (accelerometer OR accelerometry OR “objectively measured sedentary” OR “objectively measured physical” OR device-based OR “pedometer” OR “inclinometer”) were used to search related studies by two independent investigators. To provide stronger evidence of causal temporality, only prospective cohort studies were eligible for inclusion in this review. RCTs were not included given that they may not have sufficiently long follow-up periods to accumulate sufficient fatalities. All searches were limited to English-language peer-reviewed journal articles. Additional potential articles were identified by manually checking the reference lists of included papers and searching the authors’ own literature databases. The full search strategy is show in Figure 1.

### 2.2. Study Inclusion and Exclusion Criteria

The main inclusion criteria were: (1) Older adult participants aged 65 or above; (2) prospective study design; (3) device-based measured (e.g., using wearable monitors/accelerometers) ST as an exposure variable and all-cause mortality as an outcome variable; (4) provided estimates of hazard ratio (HR) or odds ratio (OR) or relative risk (RR) with 95% confidence intervals (CIs) for all-cause mortality. The exclusion criteria included the following: (1) Study population mean age was less than 65 years; (2) did not provide cut-off point of ST and all-cause mortality; (3) studies assessing specific populations, such as people living in institutions, patients with diabetes, cancer, or cardiovascular diseases etc.

Electronic literature searching yielded 4079 studies (see Figure 1). After duplicates were removed (*n* = 480), a total of 3599 articles remained. After titles and abstracts were screened, 25 full-text articles were left for potential inclusion [8,9,11,12,13,14,15,17,18,19,20,21,22,23,24,25,26,27,28,29,30,31,32,33,34]. Of these 25 articles, six studies met the criteria [8,9,11,12,14,17]. We contacted the authors of the remaining 19 studies via email because of the following reasons: (1) when missing information was not readily available in their papers (e.g., mean age or cut-off points of total sitting time); (2) request data re-analysis due to not adjusting for moderate-to-vigorous physical activity or accelerometer wear time. Five of them provided the requested results [13,15,21,22,23]. Nine studies were excluded because they included both adult and older adult populations and the mean age of their participants was less than 65 years [18,19,20,24,26,29,32,33,34]. Five studies did not provide cut-off point of ST because data re-analysis was not available [25,27,28,30,31]. Finally, 11 peer-reviewed journal articles were eligible for the subsequent review.

Among the 11 included studies, four of them did not include accelerometer wear time or standardize wear time for individuals [8,9,14,15] and MVPA [9], and were further contacted for data re-analyses. However, only one of them provided the requested results (i.e., further adjusting for MVPA and accelerometer wear time) [9]. Although some authors of the research papers included in this review provided additional analyses for the meta-analysis, these did not involve any changes to the published findings.

### 2.3. Quality Assessment

The quality assessment of the included studies was independently examined by the two reviewers (M.-C.H. and Y.L.) using an adapted 14-item quality criteria checklist proposed by Kmet et al. [35]. The 14-item checklist consisted of assessing various aspects (e.g., ‘Question/objective sufficiently described?’ and ‘Study design evident and appropriate?’) and scored 0 for no, 1 for partial, 2 for yes answer format [35]. The score of each study is presented in online supplementary Table S1. The sum of all scores was then divided by the highest possible score, giving quality scores ranging from 0 (worst) to 1 (best), the score ≥ 0.85 was defined as high quality [36]. The quality of all identified studies was high (average = 0.98).

### 2.4. Data Extraction

All publications were examined independently by the two reviewers (M.-C.H. and Y.L.). Information was extracted including: author (s), year of publication, country, number of participants, number of deaths from all-cause mortality, age at baseline, percentage of men, length of follow-up, ST measurement, covariates included in adjusted models, cut-off of duration ST (hours/day), and the HR estimates with corresponding 95% CIs for models. Disagreements between the reviewers were settled through discussion or with the third reviewer’s (P.-W.K.) involvement.

### 2.5. Publication Bias

Publication bias was evaluated assessed by Egger’s test [37] and Duval and Tweedie’s Trim and Fill test [38]. The former test is utilized for assessing funnel plot asymmetry. If the tests provide a significant result, it means that the funnel plot is asymmetric, suggesting that publication bias may occur because small studies with small effect sizes (i.e., insignificant findings) are not published and then not included in the meta-analysis. The latter one could provide a funnel plot that comprises both the included studies and the imputed studies for examining effect size shift. If the shift is trivial, then one can be more confident in the validity of the reported effect [39].We also visually examined the funnel plots for potential asymmetry.

### 2.6. Statistical Analysis

To mitigate the potential confounding effect in each study, the maximally adjusted relative risks (i.e., HRs) from multivariable proportional hazards models were employed. All of the HRs and the corresponding CIs were transformed into the natural logarithm of the HRs and their variances for subsequent meta-regression analyses.

Categorization of ST was based on the data available from each individual study. To explore the threshold of daily ST for elevating the risk of all-cause mortality, we assigned the median or mean level of ST in each category as the “dose of ST” for the corresponding relative risk for each study. For studies reporting ST by ranges of time, we calculated the midpoint of the range in each category. When the lowest category was open-ended, the lower boundary was set to zero. When the highest category was open-ended, we assumed the length of the open-ended interval to be the same as that of the adjacent interval [40,41,42,43,44,45,46,47]. Heterogeneity between studies was evaluated using *I*^2^ (i.e., the proportion of total variation explained by variation between studies). *I*^2^ values of 25%, 50%, and 75% were regarded as low, moderate, and high levels of heterogeneity, respectively [48].

We conducted random-effects meta-regression analyses to explore the shape of the associations of ST with log-transformed risk of all-cause mortality using pooled data extracted from the 11 prospective cohort studies. In addition to a linear model being tested to determine the model of best fit for the pooled dose-response data [49], we investigated second-order fractional polynomial models to determine the model of best fit for the pooled dose-response data. These included the quadratic model and a range of possible functions such as U-shaped and J-shaped patterns, which were comprehensively examined using the model—(log HR | X) = β_1_X^P1^ + β_2_X^P2^, in which X represents sedentary time (hours per day) and β represents a meta-regression coefficient. P1 and P2 were chosen from a predefined set P = [−2, −1, −0.5, 0, 0.5, 1, 2], in which X^pi^ denotes X^pi^ if p^i^ ≠ 0 and log X if p^i^ = 0 [50,51]. The results of goodness of fit tests among the 29 models are displayed in online supplementary Table S2 and in online Supplementary Table S3. The selection of the best fit model was based on the R^2^ analog. (i.e., more variance between studies explained by the model is better) [52,53].

We performed several random-effects meta-regression models with restricted maximum likelihood estimations in the following analyses. First of all, based on all studies (*n* = 11 studies, 31 effect sizes), the 29 random-effects meta-regression models were conducted to assess the shape of the associations of ST and all-cause mortality to determine the model of best fit for the pooled dose-response data. Because the linear model was determined to be the best model, we then conducted the meta-regression based on the linear model (Model 1). Then, we conducted the meta-regression again to assess effects after excluding three studies with potential bias (since they did not adjust for accelerometer wear time), and these results are presented in Model 2 [8,14,15]. Before conducting the third model, simple meta-regression analyses were performed to identify study-level factors that could modify the association between ST and all-cause mortality and contribute to the heterogeneity across studies. Mean age, percentage of males, sample size at baseline, number of covariates, study quality scores, and mean length of follow-up were scrutinized in a simple meta-regression model. The cut-point for baseline study sample size was determined by their median (1016 participants), which is close to 1000. Except this variable, the remaining study-level factors were all included as continuous predictors. The variables reaching the significance level (*p* < 0.05) were then included in Model 3.

Because the choice of the score for the open-ended category may have a substantial impact on the results using this method [54], we followed the approach of previous meta-analyses to set the midpoint of the open-ended upper interval as 1.2 times the lower boundary [55,56,57]. Based on the same meta-regression procedures in Models 1–3, sensitivity analyses were further conducted to test the stability of the findings (Models 4–6).

The above analyses assumed that the estimates of both slope and standard error assume independence of the odds ratios across levels of duration. This may not be true because the estimates for separate exposure levels (i.e., hazard ratios) were derived from the same study [48]. To assess the clustered effects within studies, we utilized the weighted linear mixed model with random-effects based on the restricted maximum likelihood (REML) method, which took the covariance parameter estimates for the ‘study’ random effect into account. Model 2 and Model 3 in Table 2, which are based on the studies adjusting for accelerometer time, were re-analyzed.

The Knapp-Hartung method was utilized in the random-effects meta-regression analyses to adjust for the dispersion across studies and yield more accurate estimates. This method additionally uses a refined estimation of between-studies variance of the effect estimator through a Student-t distribution instead of Z-distribution [39,58]. This correction will result in wider CIs of estimates and yields more conservative inferences.

We conducted meta-regression models to produce the scatter plots with regression lines and 95% CIs to visualize the association of ST and mortality risk and identify the potential threshold of ST for each model.

All analyses were conducted using Comprehensive Meta-Analysis Version 3.3.070 (Biostat, Englewood, NJ, USA) [52] and IBM SPSS 24.0 software (International Business Machines Corporation, Armonk, New York, USA). Two-sided *p* values less than 0.05 were regarded as significant.

## 3. Results

### 3.1. Study Characteristics

The characteristics of the 11 studies included in the meta-analysis are summarized in Table 1. Data across studies involved 36,341 participants. Studies were conducted in five different countries, and mean baseline age ranged from 66.7–79.0 years. The follow-up period varied from 2.3–14.2 (mean time = 5.6) years, by which time 2200 (6.1%) participants had died.

All studies utilized accelerometers, with six studies defining ST as <100 counts/min [9,12,14,17,22,23], two studies defined it as <200 counts/min [13,21], one used <50 counts/min [11], while in two other studies, the definition of ST was not shown [8,15]. All studies adjusted for multiple potential confounding factors ranging from 9 to 18 covariates. Each study was adjusted for age and sex, while 7 out of 11 studies adjusted for body mass index (BMI), and 9 studies for education and MVPA, 10 studies for smoking, 8 studies for alcohol consumption, 4 studies for hypertension, 6 studies for diabetes and cancer each. Other covariates varied across the studies. Eight studies included adjustment for accelerometer wear time [9,11,12,13,17,21,22,23], while three did not report accelerometer wear time [8,14,15].

The heterogeneity of effect estimates among studies (*I*^2^) was 39.3%, suggesting a low-to-moderate inconsistency across the studies [59].

### 3.2. Sedentary Time and Mortality: Dose-Response Meta-Regression

A random-effects meta-regression model was first conducted to assess the shape of the associations of ST and all-cause mortality based on all studies (*n* = 11 studies, effect sizes = 31). The linear model possessed the highest value of the R^2^ analog. (0.34), and explained more variance between studies than the other 28 models (see Supplementary Table S2). However, the regression coefficient (0.04, *p* = 0.15) was not significantly different from zero (Model 1 in Table 2). These results revealed that there was not any consistent pattern of dose-response associations across studies.

Second, we conducted meta-regression after excluding the three studies without adjusting for accelerometer wear time (*n* = 8 studies, effect sizes = 24). The linear model again showed the best fit for the pooled dose-response data among the 29 models (R^2^ analog. = 0.73, see in online Supplementary Table S3). This indicated a linear dose-response relationship between daily ST and log-transformed risk of mortality (regression coefficient = 0.08, *p* = 0.02) (Model 2 in Table 2).

Third, we performed several simple meta-regressions to explore several study-level variables, such as mean age, percentage of males, sample size at baseline, number of covariates, study quality scores, and mean length of follow-up in a simple meta-regression model. Among these, only sample size reached significance (*p* < 0.05), so this was included in Model 3. The results demonstrated that studies with larger sample sizes (median of sample sizes = 1000, *n* < 1000 [12 effect sizes] vs. *n* ≥ 1000 [12 effect sizes, reference]) tended to have weaker associations between daily ST and mortality risks (see Table 2).

Fourth, to assess the potential impact of adopting this coding method for open-ended upper intervals, sensitivity analyses were conducted to test the stability of the findings based on the same meta-regression procedures in Models 1–3, (Models 4–6 in Table 2). The results were similar to those in Models 1–3, suggesting the stability of these findings. 

Finally, to assess the clustered effects within studies on the results of the Model 2 and the Model 3, the weighted linear mixed models with random-effects were conducted. The coefficients (SEs) are 0.09 (0.03) (*p* = 0.003) for the Model 2 and 0.10 (0.03) (*p* = 0.002) for the Model 3. These results are similar to those in Table 2. In addition, the covariance parameter estimates for the ‘study’ random effect in the two models were both non-significant (*p* = 0.15 for Model 2; *p* = 0.35 for Model 3), suggesting that the clustered effects within studies are small. 

### 3.3. Visual Assessment of Dose-Response Relationships

**Figure 2:**
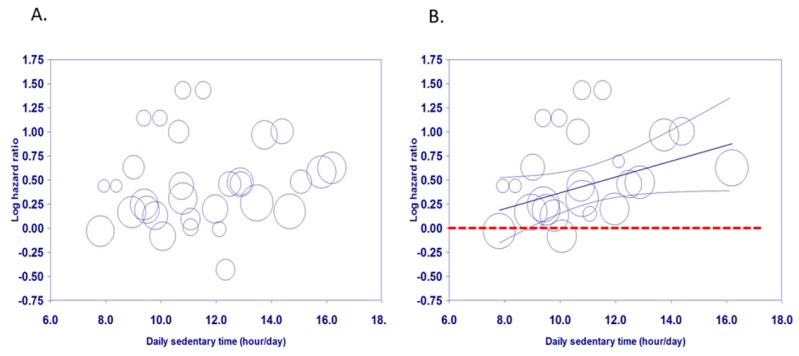
Scatter plots of meta-regression for Model 1 (**A**) based on all studies and 2 (**B**) excluding studies without adjustment for accelerometer wear time).

The scatter plot of Model 1 illustrates the association of doses of sitting time per day and log-transformed mortality risk was irregularly dispersed (Figure 2A). In contrast, the scatter plot of Model 2 demonstrates that there is a linear association between doses of daily sitting time and log-transformed mortality risk (Figure 2B). The regression line and the upper and lower lines for 95% confidence interval revealed that increased mortality risks became significant when total ST exceeded approximately 9 h/day. Figure 2 Meta-regression of all-cause mortality risk associated with daily ST based on Model 1 (A) and Model 2 (B). Each estimate is represented by a circle. The size of each circle is proportional to that estimate’s weight (i.e., 1/variance of the estimate). The center line and the upper and lower lines show the predicted values and their 95% confidence intervals.

**Figure 3:**
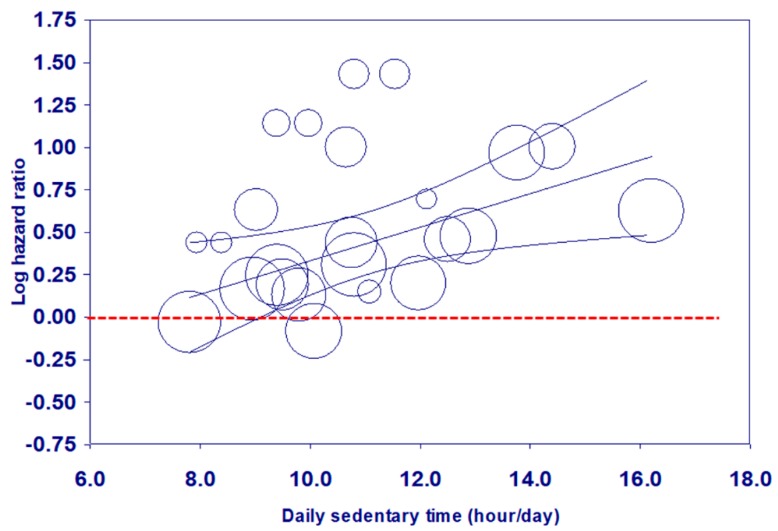
Meta-regression of all-cause mortality risk associated with daily ST based on Model 3.

The scatter plot of Model 3 (Figure 3) showed a similar pattern to Figure 2, even after adjusting for sample size at baseline. 

### 3.4. Evaluation of Publication Bias

**Figure 4:**
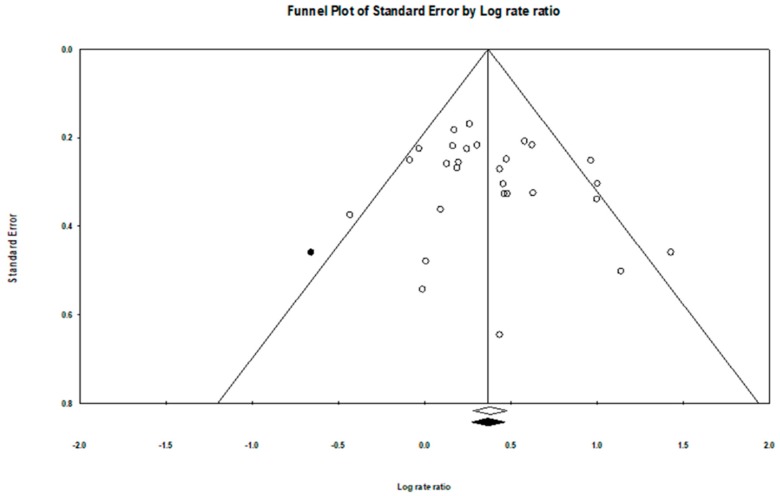
Funnel plot with imputed studies.

No significant evidence of funnel plot asymmetry was identified (Figure 4), and Egger’s test indicated that there was no evidence of publication bias (*p* = 0.08). Similarly, the observed point estimate in log unit (0.41, 95% CI = 0.28–0.54) was relatively close to the adjusted estimate after imputing 1 study (0.39, 95% CI = 0.26–0.53) in the Trim and Fill adjustment (Figure 4).

## 4. Discussion

This is the first meta-analysis to assess the relationship between objectively-assessed ST with all-cause mortality and to explore whether there is a threshold of ST above which there is an increase in mortality risk in older adults. Our meta-regression demonstrated a significant log-linear association between daily ST and all-cause mortality in older adults based on the pooled data from well-designed prospective cohort studies using objective device-based assessments. Higher amounts of time spent in sedentary behaviors were associated with elevated mortality risks in older adults. These findings were derived from studies that adjusted for multiple confounders, and the sensitivity analyses provided further support for the robustness of effects.

There was a log-linear association between daily ST and mortality, supporting previous systematic reviews and meta-analysis for adults or the general populations [6,60]. However, the log-linear association was only found after excluding studies that did not adjust for accelerometer wear time. Failure to take absolute wear time into account means that estimates of ST may vary according to the duration of objective recordings. This effect may have implications for future systematic reviews or meta-analyses based on device-based assessment of ST.

This study indicated that more time spent in sedentary behaviors was related to elevated mortality risks in older adults, which supports the findings of a recent narrative review [6]. The bubble plots and the regression lines and their 95% confidence intervals in Figure 2 and Figure 3 revealed that increased mortality risks became significant when total ST exceeded approximately 9 h/day, which is the same as the cut-off for adults aged 18–64 years found in our recent analysis [60]. However, the cut-points for the categories, including the reference group were not consistent across the included studies. The hazard ratios derived from each study are relative rather than absolute. Based on a limited number of studies, the results from this meta-analysis only offer a starting point for considering a cut-off of daily sedentary time. Public health and clinical recommendations should continue to advise older adults to spend less time in prolonged sitting.

Unfortunately, the weighted average of ST using objective measures is 10.08 h/day, which is higher than that in the general adult population of 8.65 h/day [61]. This means that more than half of the elderly population is at risk of elevated mortality. Additionally, older people spending more time in sedentary behaviors showed progressive increases in mortality risk from a HR of 1.33 to 1.45 (10 h/day), 1.70 (12 h/day) and 2.01 (14 h/day) (data not shown). 

The log-linear relationships of daily ST with mortality risks were further supported by the sensitivity analysis that controlled for sample size at baseline. Studies with a larger sample size tended to report weaker relationships between daily ST and death hazards. There is no clear explanation for this result. It may be related to publication bias against non-significant findings, in that effect sizes reported in meta-analyses may be negatively correlated with study sample sizes [62]. However, publication bias was checked, which was not significant.

Short periods of follow-up raise the possibility of reverse causation, with illnesses that precede death limiting people’s physical activity. However, several studies included in this meta-analysis have observed similar results after excluding early deaths in the first year of follow-up [13] or excluding those with mobility disability and prevalent cardiovascular diseases [12], suggesting that the findings may be not due to reverse causation.

A recent review suggested that the associations between sitting time and all-cause mortality may be dependent on MVPA levels, rather than being direct effects of sedentary behavior [6]. An authoritative meta-analysis demonstrated that individuals who engage in MVPA for about 60–75 min per day do not show an increased risk of mortality even if they spend more than 8 h a day sitting [36]. However, these findings were mainly based on studies assessing ST in general populations by self-report, and did not focus on older adults.

This meta-analysis has several strengths. First, it is the first meta-regression exploring the dose-response relationships between ST and mortality risks in older adults based on high quality cohort studies with objectively-assessed measures; these can provide more accurate estimation of daily ST and its relation with mortality than studies based on self-report. Second, the pooled data allowed us to examine whether these is a potential threshold of ST above which hazard of dying is elevated more precisely than any single study or meta-analysis with self-report measures. Finally, official death registry records provided high quality data for mortality ascertainment.

The main limitation of this meta-analysis is that findings are based on a limited number of studies. Second, although we adopted the maximally adjusted hazard risks that took into account the main likely confounders such as age, sex, educational attainment, health behaviors and health conditions, MVPA and accelerometer wear time, the issue of unmeasured confounding cannot be ruled out [63]. It would be desirable if factors such as physical or social environment attributes were taken into account in future studies. In contrast, over-adjustment bias, which implies that control for an intermediate variable on a causal path from exposure to outcome [64], was not dealt with or reported in the articles reviewed. Therefore, the potential bias of inappropriate adjustment cannot be ignored when interpreting the results. Third, different studies adopted differential criteria for defining and computing ST, which may have led to misclassification. Fourth, all included studies were conducted in the United States or Europe. Therefore, the results may not be generalizable to other populations. Fifth, our literature search was limited to the English language. These publications may not represent all of the evidence. Finally, the current meta-regression was conducted using all-cause mortality as the outcome. These findings may be not generalizable to other health outcomes such as cardiovascular diseases or diabetes.

## 5. Conclusions

In summary, it has been argued that the current evidence concerning sedentary behaviors has not been sufficient to inform quantitative public health guidelines due to the lack of long-term prospective studies using objective measures of ST [6]. Our meta-regression suggests that there is a log-linear dose-response association between daily ST and all-cause mortality in older adults. Higher amounts of time spent in daily sedentary behaviors were associated with elevated mortality risks in older adults. These results provide preliminary evidence for considering a cut-off of daily sedentary time and suggest that older people spend less time in sedentary behaviors. Future studies are encouraged to conduct dose-response meta-regression based on individual participant data (IPD) to verify these findings. We hope these findings will provide additional evidence that supports the development of public health guidelines on prolonged sitting.

## Figures and Tables

**Table 1:** Characteristics of studies.

Author (Year), Country	Study Population			Follow-Up Years Mean	ST Measure (Mean or Median Time)	Covariates (Number of Covariates)	Cut-off (h/d)	Cox Regression HR (95% CIs)	Quality
	*n* (Death)	Age Mean (±SD)	Male (%)						
Koster et al., 2012, USA [22]	1091 (126)	≥65 *M* = 74.7 (±6.5)	68.8%	2.8 y	Objectively measured SB < 100 counts/1 min [AM-7164 ActiGraph] (ST median: male = 9.18, female = 8.68 h)	Age, sex, race/ethnicity, education, BMI, diabetes, coronary heart disease, congestive heart failure, cancer, stroke, mobility limitation, smoking, alcohol, MVPA, accelerometer wear time (15)	Quartile: male/female		1.0
							<7.6/<7.2 (ref.)	1.00	
							>7.6–≤9.18/ > 7.2–≤8.68	1.55 (0.44–5.54)	
							>9.18–≤10.75/ >8.68–≤10.09	3.13 (1.17–8.39) *	
							>10.75/>10.09	4.18 (1.70–10.31) *	
Ensrud et al., 2014, USA [15]	2918 (409)	≥71 *M* = 79 (±5.2)	100%	4.5 y	Objectively measured SB ≤ 1.50 METs [sensewear pro armband] (ST median = 14.08 h)	Age, sex, race, education, marital status, site, season, health status, comorbidity burden, depressive symptoms, cognitive function, body fat %, number of instrumental activity of daily living impairments, smoking, sleep time, gait speed, self-reported total PA (17) ^¶^	≤12.86 (ref.)	1.00 ^†^	0.95
							12.87–14.08	1.30 (0.93–1.81)	
							14.09–15.24	1.19 (0.83–1.70)	
							>15.24	1.79 (1.19–2.70) *	
^§^ Fox et al., 2015, UK [9]	208 (32)	≥70 *M* = 78 (±5.7)	51.2%	4.3 y	Objectively measured SB < 100 counts/1 min [Actigraph GT1Ms] (ST median = 11.03 h)	Age, sex, educational, index of multiple deprivation, weight status, general practitioner system, number of self-reported chronic illnesses at baseline, lower limb function (9) ^¶^; Re-analysis further including MVPA & accelerometer wear time = 11)	<10.55 (ref.)	1.00	1.0
							≥10.55–11.59	1.01 (0.39–2.56)	
							≥11.6	0.99 (0.34–2.86)	
Schmid et al., 2015, USA [14]	1677 (112)	≥50 *M* = 67.2 ^‡^	49%	2.9 y	Objectively measured SB < 100 counts/1 min [AM-7164 ActiGraph] (ST median = 8.6 h)	Age, sex, education, ethnicity, history of diabetes, cardiovascular disease, cancer, mobility limitations, BMI, smoking, alcohol, light PA, MVPA (13) ^¶^	<8.60 (ref.)	1.00	0.95
							≥8.60	1.59 (0.84–3.03) ^††^	
Lee, 2016, USA [23]	1768 (453)	≥65 *M* = 74.7 (±6.5)	51.8%	6.3 y	Objectively measured SB < 100 counts/1 min [AM-7164 ActiGraph] (ST median = 10.0 h)	Age, sex, education, income, BMI, self-reported general health, condition, high blood pressure, high cholesterol, type 2 diabetes, history of heart attack, stroke, cancer, energy intake by 24-h dietary recall, binge drinking, smoking, MVPA, accelerometer wear time (18)	Quartile		1.0
							≥4.6–<8.8 (ref.)	1.00	
							≥8.8–<10.0	1.28 (0.82–1.99)	
							≥10.0–<11.6	1.36 (0.89–2.09)	
							≥11.6–<20.8	1.87 (1.22–2.86) *	
Klenk et al., 2016, Southern Germany [8]	1271 (100)	≥65 *M* = 75.6 (±6.51)	46.4%	4 y	Objectively measured lying or sitting activPAL (ST median = 11.75 h)	Age, sex, education, BMI, diabetes, hypertension, cardiovascular disease, cancer, chronic kidney disease, blood glucose. smoking, alcohol, walking time (which includes light, moderate and vigorous intensity physical activity) (13) ^¶^	5.85–<10.4 (ref.)	1.00	0.95
							≥10.4–<11.75	1.10 (0.54–2.24)	
							≥11.75–<12.94	0.65 (0.31–1.35)	
							≥12.94–<17.21	1.62 (0.85–3.07)	
Diaz et al., 2017, USA [11]	7985 (340)	≥45 *M* = 69.8 ^‡^	45.8%	4 y	Objectively measured SB < 50 counts/1 min [Actical-Philips Respironics] (ST median = 12.44 h)	Age, sex, race, region of residence, education, season, BMI, diabetes, hypertension, dyslipidemia, estimated glomerular filtration rate < 60 mL/min/1.73 m^2^, atrial fibrillation, history of coronary heart disease, stroke, smoking, alcohol, MVPA, standardized 16 h of accelerometer wear (18)	<11.50 (ref.)	1.00 ^†^	1.0
							≥11.50–<12.44	1.22 (0.74–2.02)	
							≥12.44–<13.32	1.61 (0.99–2.63)	
							≥13.32	2.63 (1.60–4.30) *	
Dohrn et al., 2017, Sweden [17]	851 (79)	≥35 *M* = 66.7 (±10.2)	44.1%	14.2 y	Objectively measured SB < 100 counts/1 min [AM-7164ActiGraph] (ST mean = 8.20 h)	Age, sex, education, hypertension, heart disease, cancer, diabetes, BMI, smoking, MVPA, accelerometer wear time (11)	6.55–<8.20 (ref.)	1.00	1.0
							8.20–<9.83	1.88 (0.99–3.55)	
							≥9.83	2.72 (1.40–5.30) *	
Koolhaas et al., 2017, The Netherlands [21]	650 (148)	65–98 *M* = 72.6 ^a^	47.4%	11 y	Objectively measured SB ≤ 199 counts/1 min [Actiwatch model AW4] (ST mean = 9.28 h [1.86]; median = 9.20 h)	Age, sex, education, number of comorbidities, the 24 h activity rhythm, activities of daily living score, smoking, alcohol, MVPA, cohort and time awake (10)	<8 (ref.)	1.00	0.95
							8–<11	1.21 (0.71–2.04)	
							≥11	1.58 (0.87–2.88)	
Jefferis et al., 2018, UK [12]	1181 (194)	71–92 *M* = 78.4 (±4.6)	100%	5.0 y	Objectively measured SB < 100 counts/1 min [ActiGraph GT3X] (ST mean = 10.3 h)	Age, sex, region of residence, living alone, season of wear, social class, BMI, mobility disability, alcohol, smoking, sleep time, MVPA, accelerometer wear time (13)	4.9–<9.3 (ref.)	1.00	1.0
							≥9.3–<10.3	1.14 (0.69–1.91)	
							≥10.3–<11.2	1.55 (0.91–2.64)	
							≥11.2–<17.6	2.73 (1.50–4.95) *	
Lee et al., 2018, USA [13]	16,741 (207)	*M* = 72.0 (±5.7)	0%	2.3 y	Objectively measured SB < 200 counts/1 min [ActiGraph Corp] (ST mean = 8.4 h)	Age, sex, hormone therapy, parental history of myocardial infarction, family history of cancer, general health, cardiovascular disease, cancer, cancer screening, smoking, alcohol, intakes of saturated fat/fiber/fruits/ vegetables, MVPA, accelerometer wear time (14)	<7.24 (ref.)	1.00	1.0
							≥7.24–<8.38	0.97 (0.62–1.50)	
							≥8.38–<9.51	1.18 (0.77–1.82)	
							≥9.51	0.92 (0.56–1.50)	
Average of total *n* (death) = 3303 (200) Total *n* = 36,341/ Deceased *n* = 2200		Total sample *M* (±SD) age = 73.5 (±4.3) y Total follow-up *M* (±SD) year = 5.6 (±3.7) y			Total weighted average of ST = 10.08 h				*M* = 0.98

* *p* < 0.05; *p* for trend. ^†^: significant (*p* < 0.05); ^‡^: Two studies did not report mean age of the study samples. The mean age of these studies was recalculated as follows: ∑ (median age of an age group) × (sample size of a age group) divided by the total sample size [11,14]. ^a^: One study reported mean age but did not provide SD of sample age [21]; ^§^: After further adjusting MVPA and accelerometer wearing, the HRs (95% CI) for Fox’s study were recalculated as follows: <10.55 h: 1.00 (ref.); 10.55–11.59 h: 1.16 (0.37–3.65); ≥11.6 h: 2.00 (0.53–7.58); ^¶^: covariates without including MVPA or accelerometer wear time; ^††^: The results excluding deaths that occurred during the first year of follow up. Abbreviations: BMI, body mass index; *M*, mean; y, years; HR, hazard ratio; CIs, confidence intervals; h/d, hour/day; PA, physical activity; MVPA, moderate to vigorous physical activity; SD, standard deviation; ST, sedentary time.

**Table 2:** Dose-response relationships of objectively-measured sedentary time with all-cause mortality assessed using random-effects meta-regression models.

Models	Number of ES	Coefficients (SE)	t	*p* Value
Model 1	31			
Sedentary time		0.04 (0.03)	1.49	0.15
Model 2	24			
Sedentary time		0.08 (0.03)	2.49	0.02
Model 3	24			
Sedentary time		0.10 (0.03)	3.65	0.002
Sample size (*n*) (≥1000 = 1 vs. <1000)		−0.43 (0.14)	−3.16	0.01
Model 4 (sensitivity analysis 1)	31			
Sedentary time		0.04 (0.02)	1.91	0.07
Model 5 (sensitivity analysis 2)	24			
Sedentary time		0.08 (0.03)	3.12	0.01
Model 6 (sensitivity analysis 3)	24			
Sedentary time		0.09 (0.02)	3.84	0.001
Sample size (*n*) (≥1000 = 1 vs. <1000)		−0.41 (0.14)	−2.96	0.01

ES, effect size; SE, standard error. t: Knapp-Hartung method; Models 2 and 3 and Models 5 and 6, excluding studies without adjustment for accelerometer wear time.

## Supplementary Materials

The following are available online at https://www.mdpi.com/2077-0383/8/4/564/s1, Table S1: Quality assessment of systematic reviews by Kmet, Lee and Cook rating; Table S2: Goodness of fit for meta-regression analysis based on Model 1; Table S3: Goodness of fit for meta-regression analysis based on Model 2.
