# Supplementary material for: A Threshold of Objectively-Assessed Daily Sedentary Time for All-Cause Mortality in Older Adults: A Meta-Regression of Prospective Cohort Studies
Source: J Clin Med. 2019 Apr 25;8(4):564. doi: 10.3390/jcm8040564 (PMC6517908; doi:10.3390/jcm8040564)
Supplement: Supplementary file 1 [file jcm-08-00564-s001.pdf]

Journal of
Clinical Medicine

MDPI

Table S1. Quality assessment of systematic reviews by Kmet, Lee and Cook rating.

**Author (Year)**	**Assessment items**														**Overall rating**
	**1**	**2**	**3**	**4**	**5**	**6**	**7**	**8**	**9**	**10**	**11**	**12**	**13**	**14**	
Koster et al., 2012	Yes<br>Yes	Yes<br>Yes	Yes<br>Yes	Yes<br>Yes	N/A<br>N/A	N/A<br>N/A	N/A<br>N/A	Yes<br>Yes	Yes<br>Yes	Yes<br>Yes	Yes<br>Yes	Yes<br>Yes	Yes<br>Yes	Yes<br>Yes	1.0
Lee, 2016	Yes<br>Yes	Yes<br>Yes	Yes<br>Yes	Yes<br>Yes	N/A<br>N/A	N/A<br>N/A	N/A<br>N/A	Yes<br>Yes	Yes<br>Yes	Yes<br>Yes	Yes<br>Yes	Yes<br>Yes	Yes<br>Yes	Yes<br>Yes	1.0
Diaz et al., 2017	Yes<br>Yes	Yes<br>Yes	Yes<br>Yes	Yes<br>Yes	N/A<br>N/A	N/A<br>N/A	N/A<br>N/A	Yes<br>Yes	Yes<br>Yes	Yes<br>Yes	Yes<br>Yes	Yes<br>Yes	Yes<br>Yes	Yes<br>Yes	1.0
Dohrn et al., 2017	Yes<br>Yes	Yes<br>Yes	Yes<br>Yes	Yes<br>Yes	N/A<br>N/A	N/A<br>N/A	N/A<br>N/A	Yes<br>Yes	Yes<br>Yes	Yes<br>Yes	Yes<br>Yes	Yes<br>Yes	Yes<br>Yes	Yes<br>Yes	1.0
Koolhaas et al., 2017	Yes<br>Yes	Yes<br>Yes	Yes<br>Yes	Yes<br>Yes	N/A<br>N/A	N/A<br>N/A	N/A<br>N/A	Yes<br>Yes	Yes<br>Yes	Yes<br>Yes	Yes<br>Yes	Partial<br>Partial	Yes<br>Yes	Yes<br>Yes	0.95
Lee et al, 2018	Yes<br>Yes	Yes<br>Yes	Yes<br>Yes	Yes<br>Yes	N/A<br>N/A	N/A<br>N/A	N/A<br>N/A	Yes<br>Yes	Yes<br>Yes	Yes<br>Yes	Yes<br>Yes	Yes<br>Yes	Yes<br>Yes	Yes<br>Yes	1.0
Jefferis et al., 2018	Yes<br>Yes	Yes<br>Yes	Yes<br>Yes	Yes<br>Yes	N/A<br>N/A	N/A<br>N/A	N/A<br>N/A	Yes<br>Yes	Yes<br>Yes	Yes<br>Yes	Yes<br>Yes	Yes<br>Yes	Yes<br>Yes	Yes<br>Yes	1.0
Ensrud et al., 2014	Yes<br>Yes	Yes<br>Yes	Yes<br>Yes	Yes<br>Yes	N/A<br>N/A	N/A<br>N/A	N/A<br>N/A	Yes<br>Yes	Yes<br>Yes	Yes<br>Yes	Yes<br>Yes	Partial<br>Partial	Yes<br>Yes	Yes<br>Yes	0.95
Fox et al., 2015	Yes<br>Yes	Yes<br>Yes	Yes<br>Yes	Yes<br>Yes	N/A<br>N/A	N/A<br>N/A	N/A<br>N/A	Yes<br>Yes	Yes<br>Yes	Yes<br>Yes	Yes<br>Yes	Yes<br>Yes	Yes<br>Yes	Yes<br>Yes	1.0
Schmid et al., 2015	Yes<br>Yes	Yes<br>Yes	Yes<br>Yes	Yes<br>Yes	N/A<br>N/A	N/A<br>N/A	N/A<br>N/A	Yes<br>Yes	Yes<br>Yes	Yes<br>Yes	Yes<br>Yes	Partial<br>Partial	Yes<br>Yes	Yes<br>Yes	0.95
Klenk et al., 2016	Yes<br>Yes	Yes<br>Yes	Yes<br>Yes	Yes<br>Yes	N/A<br>N/A	N/A<br>N/A	N/A<br>N/A	Yes<br>Yes	Yes<br>Yes	Yes<br>Yes	Yes<br>Yes	Partial<br>Partial	Yes<br>Yes	Yes<br>Yes	0.95
															Mean = 0.98

All items were scored as "Yes (2)", "Partial (1)", "No (0)", "N/A=Not Applicable". The quality checklist comprises the following items: 1. Question/objective sufficiently described; 2. Study design evident and appropriate; 3. Method of subject/comparison group selection of information/input variables described and appropriate; 4. Subject (and comparison group, if applicable) characteristics sufficiently described; 5. If interventional and random allocation was possible, was it described; 6.If interventional and blinding of investigators was possible, was it reported; 7. If interventional and blinding of subjects was possible, was it reported; 8. Outcome and (if applicable) exposure measure(s) well defined and robust to measurement /misclassification bias? Means of assessment reported; 9. Sample size appropriate; 10. Analytic methods described/justified and appropriate; 11. Some estimate of variance is reported for the main results; 12. Controlled for confounding; 13. Result reported in sufficient detail; 14. Conclusions supported by the results? a: Re-calculating quality score after further adjusting MVPA and accelerometer wear time.

Table S2. Goodness of fit for meta-regression analysis based on Model 1.

Models	P-values of regression coefficients				R^2 analog.
	P1	*P*-value	P2	*P*-value	
**Linear**					
	1	0.15			0.34
**2nd-order fractional polynomials**					
	-2	0.86	-2	0.92	0.22
	-2	0.81	-1	0.93	0.22
	-2	0.77	-0.5	0.95	0.22
	-2	0.73	0	0.97	0.22
	-2	0.70	0.5	0.99	0.22
	-2	0.66	1	0.99	0.22
	-2	0.60	2	0.96	0.22
	-1	0.79	-1	0.87	0.22
	-1	0.80	-0.5	0.86	0.22
	-1	0.77	0	0.88	0.22
	-1	0.73	0.5	0.90	0.22
	-1	0.70	1	0.93	0.22
	-1	0.64	2	0.96	0.21
	-0.5	0.58	-0.5	0.83	0.22
	-0.5	0.79	0	0.85	0.22
	-0.5	0.75	0.5	0.87	0.21
	-0.5	0.72	1	0.89	0.21
	-0.5	0.66	2	0.93	0.21
	0	0.77	0	0.81	0.21
	0	0.77	0.5	0.83	0.21
	0	0.74	1	0.85	0.21
	0	0.68	2	0.89	0.20
	0.5	0.77	0.5	0.80	0.21
	0.5	0.76	1	0.82	0.20
	0.5	0.70	2	0.85	0.20
	1	0.75	1	0.78	0.20
**Squared**	1	0.72	2	0.82	0.20
	2	0.73	2	0.76	0.19

Table S3. Goodness of fit for meta-regression analysis based on Model2.

Models	P-values of regression coefficients				R^2 analog.
	P1	*P*-value	P2	*P*-value	
**Linear**					
	1	0.02			0.73
**2nd-order fractional polynomials**					
	-2	0.89	-2	0.99	0.66
	-2	0.77	-1	0.98	0.66
	-2	0.68	-0.5	0.99	0.66
	-2	0.59	0	0.99	0.66
	-2	0.49	0.5	0.99	0.66
	-2	0.41	1	0.99	0.66
	-2	0.28	2	0.97	0.65
	-1	0.70	-1	0.86	0.66
	-1	0.71	-0.5	0.82	0.66
	-1	0.60	0	0.81	0.66
	-1	0.50	0.5	0.79	0.66
	-1	0.42	1	0.78	0.66
	-1	0.28	2	0.76	0.66
	-0.5	0.29	-0.5	0.69	0.66
	-0.5	0.59	0	0.69	0.66
	-0.5	0.49	0.5	0.68	0.66
	-0.5	0.41	1	0.67	0.66
	-0.5	0.27	2	0.65	0.67
	0	0.50	0	0.58	0.66
	0	0.48	0.5	0.57	0.67
	0	0.40	1	0.56	0.67
	0	0.27	2	0.55	0.68
	0.5	0.44	0.5	0.48	0.67
	0.5	0.39	1	0.47	0.67
	0.5	0.26	2	0.46	0.68
	1	0.35	1	0.39	0.68
**Squared**	1	0.26	2	0.38	0.69
	2	0.22	2	0.25	0.70
